# Sex-Dimorphic Behavioral Alterations and Altered Neurogenesis in U12 Intron Splicing-Defective *Zrsr1* Mutant Mice

**DOI:** 10.3390/ijms20143543

**Published:** 2019-07-19

**Authors:** Francisco Alén, Isabel Gómez-Redondo, Patricia Rivera, Juan Suárez, Priscila Ramos-Ibeas, Eva Pericuesta, Raul Fernández-González, Serafín Perez-Cerezales, Keiko Horiuchi, Laura Orio, Fernando Rodriguez de Fonseca, Alfonso Gutiérrez-Adán

**Affiliations:** 1 Departamento de Psicobiología, Facultad de Psicología, Universidad Complutense de Madrid, Campus de Somosaguas, 28224 Madrid, Spain; 2 Departamento de Reproducción Animal, INIA, Avda Puerta de Hierro n° 12, Local 10, 28040 Madrid, Spain; 3 UGC Salud Mental, Instituto de Investigación Biomédica de Málaga (IBIMA), Universidad de Málaga-Hospital Universitario Regional de Málaga, Avda, Carlos Haya 82, Pabellón de Gobierno, 29010 Málaga, Spain; 4 Department of Protein-Protein Interaction Research, Institute for Advanced Medical Sciences, Nippon Medical School, 1-396 Kosugi-cho, Nakahara-ku, Kawasaki, Kanagawa 211-8533, Japan

**Keywords:** *Zrsr1*, U12 introns, hypothalamus, behavior, social interaction, neurogenesis, cell proliferation

## Abstract

Mutant mice with respect to the splicing factor *Zrsr1* present altered spermatogenesis and infertility. To investigate whether *Zrsr1* is involved in the homeostatic control that the hypothalamus exerts over reproductive functions, we first analyzed both differential gene and isoform expression and alternative splicing alterations in *Zrsr1* mutant (*Zrsr1^mu^*) hypothalamus; second, we analyzed the spontaneous and social behavior of *Zrsr1^mu^* mice; and third, we analyzed adult cell proliferation and survival in the *Zrsr1^mu^* hypothalamus. The *Zrsr1^mu^* hypothalamus showed altered expression of genes and isoforms related to the glutathione metabolic process, synaptonemal complex assembly, mRNA transport, and altered splicing events involving the enrichment of U12-type intron retention (IR). Furthermore, increased IR in U12-containing genes related with the prolactin, progesterone, and gonadotropin-releasing hormone (GnRH) reproductive signaling pathway was observed. This was associated with a hyperactive phenotype in both males and females, with an anxious phenotype in females, and with increased social interaction in males, instead of the classical aggressive behavior. In addition, *Zrsr1^mu^* females but not males exhibited reduced cell proliferation in both the hypothalamus and the subventricular zone. Overall, these results suggest that *Zrsr1* expression and function are relevant to organization of the hypothalamic cell network controlling behavior.

## 1. Introduction

The paralogous splicing factors ZRSR1 (also known as U2af1-rs1) and ZRSR2 (also known as U2af1-rs2), encoded by the *Zrsr1* and *Zrsr2* genes, have been identified in all mammalian species analyzed [1,2] and are known to recognize the 3′AG splice site of the intron. They have been attributed an essential role in splice site recognition of U12 minor introns [3]. Minor class or U12-dependent spliceosomes remove U12-type introns (<0.4% of all introns) [4], which are non-randomly distributed across the genome and, despite their scarce abundance, are highly conserved across distantly related eukaryotic taxa, indicating their evolutionary origin. Murine *Zrsr1* is a retrotransposed copy of X-linked *Zrsr2* (located on the X chromosome in all mammalian species analyzed). *Zrsr1* is located within an intron of *Commd1* and is paternally expressed in the placenta and some adult tissues, while the maternal copy is methylated and silent. *Zrsr1* knockout mice do not show an abnormal phenotype [5], probably because of the expression of the other paralog. However, we recently demonstrated that *Zrsr1* plays critical roles in hematopoiesis, muscle strength, and spermatogenesis in mice. *Zrsr1* mutation produced critical alterations at the spermatocyte stage, affecting U12 introns [1]. In contrast, female mice with mutant *Zrsr1* do not show any effect in the female estrous cycle; they are normal cyclin females and respond to superovulation like control females [1]. *Zrsr1* expression has been reported in neonatal and adult mouse brain [1,6], with the hypothalamus being the area with greatest expression (BIOGPX.org: http://ds.biogps.org/?gene=22183). Extensive genetic regulation of transcription and alternative splicing have been reported in the hypothalamus, and a considerable proportion of the new isoforms and transcripts are significantly correlated to physiological phenotypes [7]. Since the hypothalamus plays a key role in male reproductive control, in the present study we analyzed the alterations in gene expression and RNA alternative splicing in the *Zrsr1* mutant (*Zrsr1^mu^*) hypothalamus.

Genes containing U12 introns are over-represented in functions and pathways related to development, such as RNA processing, DNA replication, cell cycle, MAPK signaling, voltage-gated ion channels, signal transduction, and DNA damage repair [8,9]. Dysregulation of the minor spliceosome generally leads to intron retention and, less frequently, to exon skipping [10] and has been associated with multiple diseases, including developmental disorders [1,8,11,12,13], neurodegeneration [14], and cancer [15]. These diseases can be caused by mutations within both the protein and small nuclear ribonucleic acid (snRNA) components of the minor spliceosome. In addition, the minor spliceosome is thought to play an important developmental role in plants [16,17], *Drosophila* [18], and zebrafish [19] and in the mouse central nervous system [20]. However, the specific roles of minor splicing on the hypothalamus have not been fully elucidated. In relation with the enrichment of U12 genes related to ion channels, it is important to indicate that ion channels play essential roles in various cellular functions in the nervous system [21], and alternative splicing of ion channels induces changes in their cellular distribution, activation/inactivation kinetics, voltage/Ca2+ dependence, and pharmacological drug sensitivities [22]. 

To examine the role of the ZRSR1 minor splicing factor in the hypothalamus, we analyzed differential gene and isoform expression and alterations in alternative splicing in the *Zrsr1^mu^* hypothalamus. Furthermore, we analyzed the serum testosterone levels and social behavior of *Zrsr1^mu^* mice, and we have quantified cell proliferation and survival in the *Zrsr1^mu^* hypothalamus and subventricular zone, a major adult neurogenesis niche in rodents. Collectively, this study identifies *Zrsr1* as an essential factor for efficient U12 intron splicing in the hypothalamus, critical for the organization of hypothalamic cell networks that organize behavior.

## 2. Results

### 2.1. Expression of ZRSR1^mu^ in the Hypothalamus

**Figure 1:**
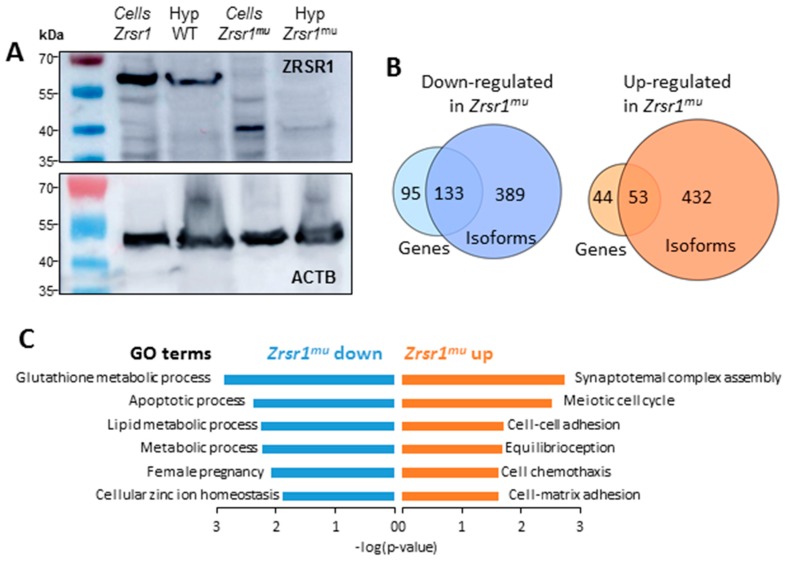
ZRSR1 expression in HEK293 murine cell lines and in mouse hypothalami. Numbers of differentially expressed genes (DEG) and differentially expressed isoforms (DEI). Significantly enriched Gene Ontology (GO) terms (*p* < 0.05) in DEGs. (**A**) Western blot analysis of ZRSR1 protein expression. Lanes 1 and 3 correspond to the murine cell lines expressing m*Zrsr1* and m*Zrsr1* truncated protein, respectively. Expression of ZRSR1 in wild-type (WT) mouse hypothalamus and *Zrsr1^mu^* mouse hypothalamus is shown in lanes 2 and 4. Actin was used as the loading control (lower panel). (**B**) Venn diagram showing the correspondence between up- and down-regulated genes and isoforms in the comparison between *Zrsr1^mu^* and WT mouse hypothalami. (**C**) Significant GO terms in DEGs in the pairwise comparison.

In order to verify the effective mutation of ZRSR1 in the hypothalamus, we conducted Western blot in wild-type (WT) and *Zrsr1^mu^* mice (Figure 1A). We detected the expression of the endogenous ZRSR1 protein at the expected molecular weight in the hypothalamus of WT mice. This band was absent in the hypothalamus of *Zrsr1^mu^* mice, demonstrating the ablation of the wild-type form of ZRSR1. Instead, we found a weak band with the expected molecular weight for the truncated protein. We conducted a number of unsuccessful attempts to enrich the truncated protein’s low signal, probably due to its short half-life, and we corroborated the molecular weight of each band using protein extracts from HEK293 stable cell lines expressing the wild-type or the mutant proteins after induction with tetracycline [1].

### 2.2. RNA-seq Analysis of Hypothalami from WT and Zrsr1^mu^ Mice

To determine the implications of ZRSR1 mutation in the hypothalamus, we performed RNA-seq on three WT and three mutant male mouse hypothalamus samples. The RNA-seq database generated is available via ArrayExpress (Accesion number E-MTAB-8012). On average, ~120 million 125 bp paired-end sequencing reads of each sample aligned uniquely against the mouse reference genome (GRCm38/mm10 version) (Supplementary Table S1). After removing genes and transcripts with low counts, we evaluated 24,466 genes for differential expression and 97,264 transcripts for differential isoform usage. With the DESeq2 R package we obtained a total of 325 genes differentially expressed (DEG) (*p*-value < 0.01) between WT and *Zrsr1^mu^* hypothalami (Supplementary Table S2). Out of those genes, 228 were down-regulated and 97 were up-regulated in *Zrsr1^mu^*. Differentially expressed isoforms (DEI) were evaluated with EBSeq software applied to transcript estimated counts obtained with RSEM software [23]. DEI analysis showed 534 isoforms (corresponding to 485 genes) that were preferentially expressed in the hypothalamus of *Zrsr1^mu^* mice, whereas the expression of 608 (522 genes) isoforms was downregulated in these animals (Supplementary Table S3). A comparison of DEGs and DEIs showed that ~57% of the up- and down-regulated genes also had altered expression of isoforms (Figure 1B, Supplementary Table S4). Nevertheless, the number of DEIs was ~3.5-fold higher than the number of DEGs. This indicates that the effect of the mutation on *Zrsr1* has a more significant impact on splicing than on transcription. Genes with lower expression in *Zrsr1*^mu^ were significantly enriched in the glutathione metabolic process, apoptotic process, metabolic processes, female pregnancy, and cellular zinc ion homeostasis Gene Ontology (GO) terms. On the contrary, genes upregulated in *Zrsr1^mu^* hypothalamus showed enrichment in synaptonemal complex assembly, meiotic cell cycle and cell processes, like cell–cell adhesion, cell chemotaxis, and cell–matrix adhesion GO terms (Figure 1C, Supplementary Table S5).

**Figure 2:**
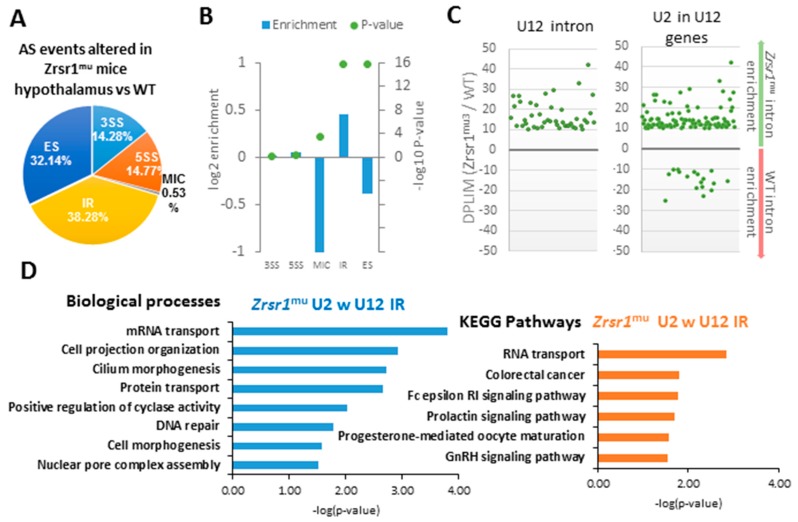
RNA-seq analysis of differential alternative splicing (AS) in *Zrsr1^mu^* mouse hypothalamus. (**A**) Distribution of the categories of AS events detected in the analysis. 3SS, alternative 3′ splice sites; A5SS, alternative 5′ splice sites; ES, exon skipping; IR, intron retention; MIC, alternative microexon. (**B**) Enrichment of the different categories of AS in our analysis in relation with the total number of events present in the vast-tools database. (**C**) Distribution of differentially retained introns that correspond to U12 introns (left) and U2 introns located in a U12-intron-containing gene (right) detected in our analysis. (**D**) GO terms associated to biological processes and KEGG pathways enriched in the U12-containing genes detected as differentially spliced.

Alternative splicing (AS) was assessed using the vast-tools sequence reference, considering a transcript as differentially spliced when its ΔPSI was higher than 0.1. We identified 1283 down-regulated events and 1776 up-regulated events in the *Zrsr1^mu^* hypothalamus, which corresponded to five different types of splicing—exon skipping (ES), alternative 3′splice site (3′ss), alternative 5′ splice site (5′ss), intron retention (IR), and microexon (MIC)—keeping only those events identified with high confidence (Supplementary Table S6, Figure 2A). Enrichment of each event was checked with a Fisher test comparing them with the total number of each event in the vast-tools database, in relation with the total number of differentially spliced events detected. All categories of AS being affected, significant enrichment (*p*-value < 0.05) was detected for MIC, ES, and IR (Figure 2B, Table 1). As *Zrsr1* has been attributed a role in both U2 and U12 minor splicing, we separated these two intron classes. Out of 665 IR events detected as upregulated in *Zrsr1^mu^*, 44 correspond to U12 introns, which represents a great enrichment when compared with the overall proportion of U12 introns in the mouse genome (~6.6% against ~0.4%). Moreover, almost 15% of the upregulated IR events occur in U2 introns located in genes with U12, which agrees with the observations in *Zrsr1^mu^* [1] and suggests a communication between both major and minor spliceosomes. The distribution of U12 and U2 introns located in U12-containing genes that show an alteration in IR between the hypothalami of *Zrsr1^mu^* and WT mice is shown in Figure 2C. 

Genes that showed IR in genes that contain U12 introns in *Zrsr1^mu^* were enriched in mRNA transport, cilium morphogenesis, protein transport, cyclase activity, DNA repair, and cell functions like cell project organization, morphogenesis, and the assembly of the nuclear pore complex (Figure 2D, Supplementary Table S7). This indicates a possible relationship between the defects observed in *Zrsr1^mu^* mice and the mis-regulation of the cell cycle. These genes also showed enrichment in mRNA transport, the FC epsilon RI signaling pathway, and prolactin, progesterone, and GnRH signaling pathway-related KEGG pathways (Figure 2D, Supplementary Table S7), suggesting an important role of *Zrsr1* in reproductive control in the hypothalamus. Because the hypothalamus and the pituitary gland are involved in the regulation of testosterone production by the testes, we analyzed serum testosterone levels in WT and *Zrsr1^mu^* mice, but no differences were observed (5.8 ± 3.9 and 4.7 ± 4.3 ng/mL, respectively). In addition, the number of Leydig and Sertoli cells was not altered in the testes of mutant mice, indicating that testosterone levels are not related with the spermatogenesis alterations previously described in these mutant mice [1].

### 2.3. Spontaneous and Social Behaviors

**Figure 3:**
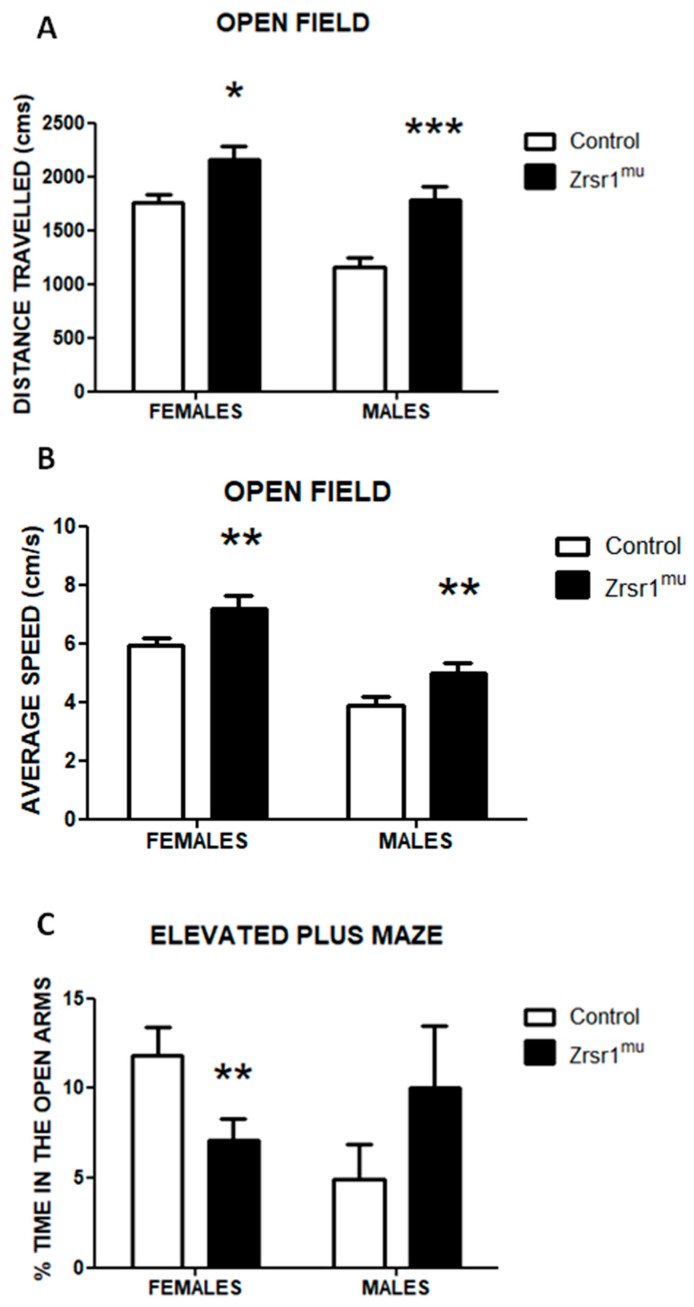
Performance in the open field test. Panel (**A**) represents total distance travelled, panel (**B**) represents average speed of movement of male and female wild-type (control) and *Zrsr1^mu^* mice. Panel (**C**) represents anxiety in the elevated plus maze of male and female wild-type and *Zrsr1^mu^* mice. Data are means ± SEM of at least 8 animals per group. * *p* < 0.05, ** *p* < 0.01, **** *p* < 0.001, 2-way ANOVA.

Because the hypothalamus is a critical region for the organization of spontaneous, social, and reproductive behaviors [24], we characterized the behavioral phenotype of *Zrsr1^mu^* mice. To this end we first analyzed spontaneous exploratory behavior in an open field test (Figure 3A,B) and in an elevated plus maze (Figure 3C). In the open field test, two-way ANOVA revealed that both *Zrsr1^mu^* males and females exhibited a clear hyperactive phenotype, reflected in both the total distance travelled in the field (Figure 3A, *F*(1,36) = 19.4, *p* < 0.001 for genotype, despite females being more active than males, *F*(1,36) = 20.8, *p* < 0.001) and the average speed exhibited (Figure 3B, *F*(1,36) = 12.6, *p* < 0.01 for genotype). This hyperactivity was also observed in the elevated plus maze (data not shown), where *Zrsr1^mu^* mice displayed clear sex-dimorphic responses in the anxiety-associated responses. Female *Zrsr1^mu^* mice showed a marked reduction in the exploration of the open arms of the maze, indicating an anxiety-like state, whereas the males exhibited an opposite non-significant trend (Figure 3C, sex × genotype interaction *F*(1,36) = 4.8, *p* < 0.04). Female mice were not controlled for estrous cycle and were randomly cycling, this factor being a potential limitation for the behavioral performance in this sex.

**Figure 4:**
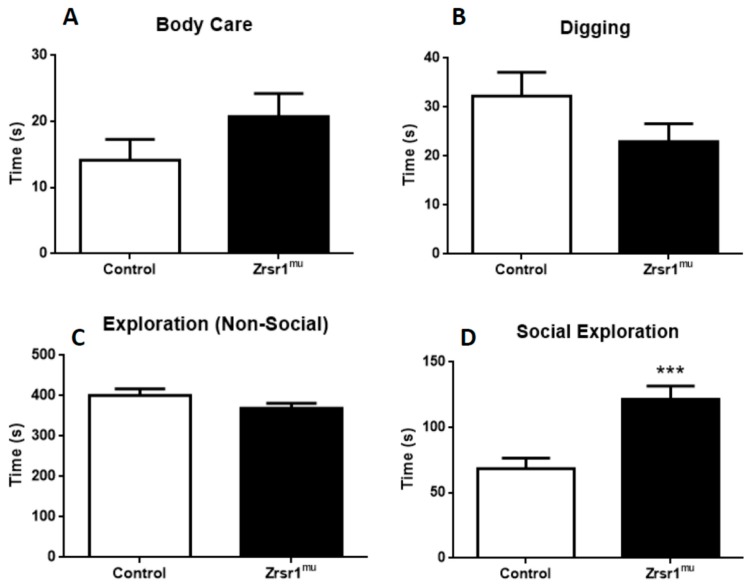
Performance in a social interaction test of male wild-type and *Zrsr1^mu^* mice. Panels: (**A**) body care activity, (**B**) exploratory digging, (**C**) non-social exploration of the field, and (**D**) time spent in social exploration. Data are means ± SEM of at least 8 animals per group. *** *p* < 0.005.

Because social interaction in males is often associated with aggressive behavior in male mice, we analyzed this pattern of behavior in a social interaction test (Figure 4). Surprisingly, neither wild-type nor *Zrsr1^mu^* mice displayed aggression, but we found a marked increase of social exploration in *Zrsr1^mu^* male mice with respect to control wild-type mice (Figure 4D, *t* = 4.02, df = 17, *p* < 0.001). 

### 2.4. Cell Proliferation and Survival

**Figure 5:**
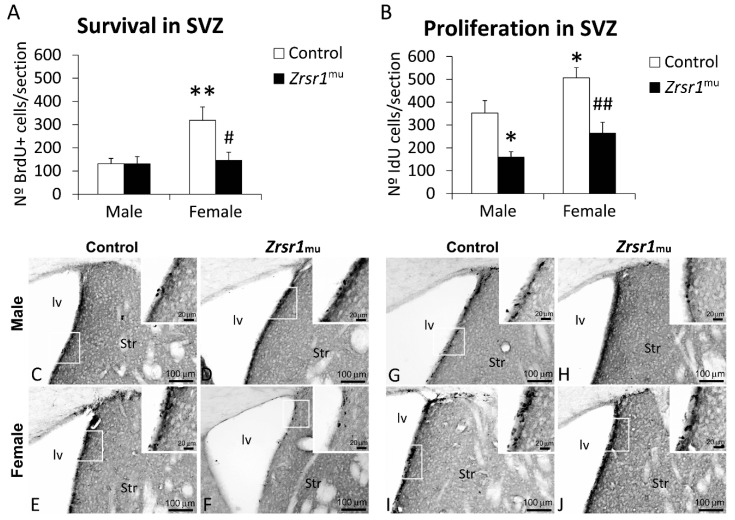
Newborn cell survival (**A**) and neural stem cell proliferation (**B**) respectively assessed by the number of BrdU- and IdU-immunoreactive (+) cells in the subventricular zone (SVZ) of male and female wild-type and Zrsr1mu mice. Low- and high-resolution photomicrographs of representative images showing BrdU+ cells (**C–F**) and IdU+ cells (G–J). Arrowheads indicate labeled nuclei. Bars represent the mean ± SEM (*n* = 7–8/group). Tukey (**A**) or simple effect analysis (**B**): * *p* < 0.05, ** *p* < 0.01 female vs. control male and # *p* < 0.05 ## *p* < 0.01 wild-type vs. *Zrsr1^mu^*. Str, striatum; lv, lateral ventricle.

**Figure 6:**
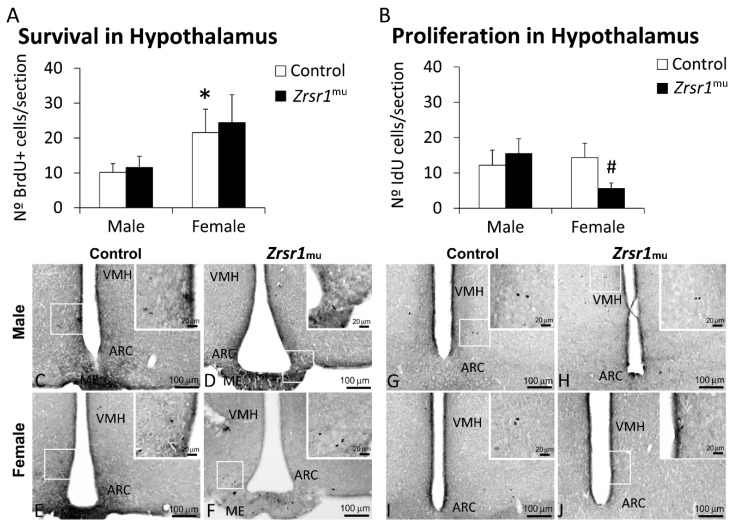
Newborn cell survival (**A**) and neural stem cell proliferation (**B**) respectively assessed by the number of BrdU- and IdU-immunoreactive (+) cells in the hypothalami of male and female wild-type and Zrsr1mu mice. Low- and high-resolution photomicrographs of representative images showing BrdU+ cells (C–F) and IdU+ cells (G–J). Arrowheads indicate labeled nuclei. Bars represent the mean ± SEM (*n* = 7–8/group). Tukey (**A**) or simple effect analysis (**B**): * *p* < 0.05 female vs. control male and # *p* < 0.05 wild-type vs. *Zrsr1^mu^*. ARC, arcuate nucleus; VMH, ventromedial hypothalamus; ME, median eminence.

The alterations found in the transcription and splicing of genes related to the cell cycle, suggesting the existence of a mis-regulation of the cell cycle, as well as alterations in transcription and/or intron retention in cell processes, led us to examine adult hypothalamic cell proliferation and survival in *Zrsr1^mu^* male and female mice. The results indicate that both male and female *Zrsr1^mu^* mice show decreased cell proliferation in the subventricular zone (*F*(1,20) = 23.7, *p* < 0.0001, Figure 5), but only females have defective cell proliferation in the hypothalamus (*F*(1,20) = 7.2, *p* = 0.014, Figure 6). Survival of proliferating cells in the subventricular zone was reduced only in female *Zrsr1^mu^* mice (sex × genotype interaction, *F*(1,20) = 4.8, *p* = 0.039, Figure 5). Overall, *Zrsr1^mu^* mice showed consistent defective cell proliferation in accordance with the alterations in gene transcription and mRNA splicing processes described above.

## 3. Discussion

The present results clearly suggest that *Zrsr1^mu^* mice have altered transcription/splicing processes in the hypothalamus and that these alterations are associated with sex-dimorphic behavioral phenotypes and adult cell proliferation processes in the brain. We performed RNA-seq on the entire hypothalamus, and this approach may result in the dilution of signals originating from specific cell populations. Nevertheless, the sensitivity of RNA-seq allows the detection of particular cell-specific markers. In fact, we could detect alterations of gene expression and AS in known markers of specific hypothalamic cell populations. Our data showed down-regulation of *Foxo3* and ES alteration in *Npy*, markers for neurons forming the arcuate nucleus. We also found up-regulation of one isoform of *Nkx2-3* and alterations in IR of *Tacc3* and ES of *Tacc3*, markers of ventromedial hypothalamus neurons (VHN); down-regulation of two isoforms of *Gfap*, a marker of astrocytes; and alteration of IR and 3′ss of *Lepr*, a marker of multiple hypothalamic cells. However, we did not observe alterations in endothelial-cell-specific genes. Interestingly, we found down-regulation of two isoforms of hemoglobin subunit alpha 2 (*Hba2*). Hemoglobin expression has also been observed in several cells, including neurons [25], and we previously reported that *Zrsr1^mu^* mice show alterations in erythrocytes [1]. We also found down-regulation of *Gnrh* (gonadotropin-releasing hormone), which is secreted by the mediobasal hypothalamus and is a major ligand of the male gonadal axis, along with down-regulation of the isoform 3 of *Fgfr3* (fibroblast growth factor receptor 3) and ES alterations of *Prok2* (prokineticin 2), two genes critical for proper gonadotropin-releasing hormone (GnRH) neuronal migration and function [26]. GnRH stimulates gonadotropins located in the anterior pituitary to secrete luteinizing hormone (LH) and follicle-stimulating hormone (FSH). FSH acts on Sertoli cells in spermatogenic tubules and LH acts on Leydig cells in the testis interstitium. The lower expression of GnRH in *Zrsr1^mu^* mice could be related with their altered spermatogenesis and infertility [1]. In addition, GnRH has been associated with the regulation of anxiety [26,27] and aggression [28] responses in rodents. GnRH signaling promotes anxiolysis, antagonizes anxiety and depression induced by corticotrophin-releasing factor, and suppresses aggression. However, central infusion of GnRH antagonists induced the opposite pattern of behavior. The behavioral phenotype of *Zrsr1^mu^* female mice (anxiety and hyperactivity) matches that of defective GnRH signaling. However, male mice exhibited an opposite pattern, with neither aggression nor anxiety and with enhanced social interaction, suggesting that other alterations in genes related with male social behavior might be affected in *Zrsr1^mu^* male mice.

In relation with the friendly, non-aggressive social interaction exhibited by male *Zrsr1^mu^* mice, it has been reported that removing testosterone and estrogens by testiclectomy in male rodents results in loss of aggression in response to territorial intrusion [29]. However, we did not find alterations in testosterone levels in *Zrsr1^mu^* mice. Although several brain areas have been implicated in aggressive behavior, little is known about male territorial aggression. The medial hypothalamus is a key component of the social decision-making network and has a long-established role in inter-male aggression in vertebrates [30]. The ventrolateral part of the hypothalamus (VMH) can mediate both acute attack and flexible seeking actions that precede attack (social perception of an opponent) [31]; silencing this area suppresses naturally occurring inter-male attack, and stimulation promotes attack [31]. Moreover, the social context exerts a dominant influence on developmentally hard-wired hypothalamus-mediated male territorial aggression [32]. Estrogen receptor alpha (ERα) levels in several brain areas, such as the anterior hypothalamus, were shown to be higher in more aggressive mice [33], and reduction of ERα in the VMH abolished aggressive behavior in males, demonstrating that expression of ERα in the VMH is necessary for aggression in adult mice [34]. In rodents, aromatase (estrogen synthetase) is expressed in the axon terminal, particularly in the hypothalamus [35]. However, we did not find differences in gene or isoform expression of genes related with testosterone or estrogens in the hypothalamus. There is also a link between glucocorticoids and aggression [36]; prevention of glucocorticoid synthesis and acute treatment with adenocorticotropic hormone (ACTH) increased fighting behavior in male mice [36]. We found down-regulation of some isoforms of several genes related with glucocorticoids, like *Sgk3* (serum/glucocorticoid-regulated kinase 3), *Crhbp* (corticotropin-releasing hormone-binding protein), and *Ucn3* (urocortin 3), in the hypothalamus of *Zrsr1^mu^* mice. These genes have been related with hormonal components of the stress response and aggressive behavior in mice [37]. Notably, MAP kinase activity has been related with social behavior circuits during resident–intruder aggression tests [38], and *Zrsr1^mu^* hypothalami presented three MAP kinases (*Mapk14*, *Mapk15*, and *Mapk8*) with U12 intron retention. Remarkably, genomic analyses have shown that minor splicing mechanisms related with cell differentiation, migration, and neural crest development had a pivotal role in the early domestication process of dogs [39].

Since the number of U12-type introns is very small, the minor spliceosome has been studied less extensively than its major counterpart, but in the last few years it has received increasing attention for its role in neurodevelopmental and neurodegenerative diseases. It has been reported that minor splicing is crucial for the survival of differentiating neurons [20], and inactivation of minor splicing in the developing mouse cortex causes self-amplifying radial glial cell death and microcephaly [40]. Our results indicated that *Zrsr1* is essential to processing U12-type introns in the hypothalamus, in the same way as in the testis [1], and that its paralogous *Zrsr2* is not able to restore the *Zrsr1* functions in these tissues. These alterations are reflected in the lower rate of cell proliferation in both the hypothalamus and the subventricular zone in female *Zrsr1^mu^* mice, and in the latter area in *Zrsr1^mu^* males. In accordance with the expected survival of differentiating neurons proposed as a result of the interference with the minor splicing system, female *Zrsr1^mu^* animals showed reduced cell survival in the subventricular zone. Overall, the alterations in cell proliferation and survival might account for the altered behavioral profile observed in *Zrsr1^mu^* mice, since adult neurogenesis (including hypothalamic) has been implicated in the organization of multiple behavioral responses, from reproductive behaviors to social/aggressive interactions [41,42].

A remarkable finding of the present study is the sex-related differences in behavior and brain cell proliferation patterns that could be related to differences in AS between sexes. A majority of the mammalian complex traits and disease phenotypes exhibit sex-related differences, but the genetic mechanisms of such differences have been largely unexplored. AS occurs frequently in the brain and impacts every step of nervous system development, including neuronal migration, cell-fate decisions, axon guidance, and synaptogenesis [43]. Sex-specific and lineage-specific changes in the expression of different splice forms have been reported [44,45]. Sex differences in splicing have been reported in the human brain [46], and a role has been suggested for sex-biased splicing in disease [46]. The sex-related alterations in behavior and brain cell proliferation due to the mutation of minor splicing factor ZRSR1 encourage further analyses of the relationship between minor splicing and gender.

## 4. Materials and Methods

### 4.1. Animals

The mouse *Zrsr1* (NM_011663.3) gene was targeted, and five transgenic lines were successfully generated [1]. For this study, two lines with 4 and 5 nt deletions were used (*Zrsr1* mutant lines 3 and 6). Homozygotes were used in all experiments. *Zrsr1^mu/+^* did not differ phenotypically from *Zrsr1^+/+^* mice, and both genotypes were used as controls. Line *Zrsr1^mu3^* in homozygosis, which is the one that presented the greatest alterations in spermatogenesis [1], was the one used for the Western blots, RNA-seq, and behavioral analysis. Transgenic mice were identified by PCR from tail DNA with primers 5´ ACGTGCTGCCGGAGTTCAAGAAC and 5´ CCTGCGTACCATCTTCCATT for amplification of the *Zrsr1* WT allele and primers 5´ CCATGACGTGCTGCCGGAGTGAAC and 5´ CCTGCGTACCATCTTCCATT for amplification of the *Zrsr1* mutant allele. All animals were of similar age (5–6 months old) and belonged to the same set of litters. RNA-seq, behavioral studies, and neurogenesis studies were done in different groups of animals. Animal experiments were carried out in strict accordance with the recommendations stipulated in the guidelines of European Community Council Directive 2010/63/EU. Experiments were approved by the Committee on the Ethics of Animal Experiments of the INIA (permit number CEEA 2012/021). For testosterone analysis, blood samples were collected from 10 WT and 10 *Zrsr1^mu^* mice and were centrifuged for serum separation, and testosterone levels in serum were determined using an ELISA kit (DRG International, Inc, Springfiel, NJ, USA) according to the manufacturer´s instructions.

### 4.2. RNA Extraction and RNA-seq Analysis

Total RNA was extracted from the hypothalami of three wild-type mice and three *Zrsr1^mu^* mice at 3 months of age using the TRIzol^®^ reagent (Invitrogen; Carlsbad, CA, USA) and then treated with DNase (Promega, Alcobendas, Madrid, Spain) for 1 h. RNA-seq libraries were prepared from the samples described above. The resulting cDNA libraries were used for sequencing using a HiSeq2500 v4 chemistry system at the Centre of Genomic Regulation (Barcelona, Spain). Sequencing yielded an average of ~65 million of 125 pb paired-end sequences per sample (~130 million sequences per sample). First, the quality of the sequences was assessed using FastQC (http://www.bioinformatics.babraham.ac.uk/projects/fastqc/). Then, the sequences were trimmed using Trimmomatic software(http://www.usadellab.org/cms/?page=trimmomatic) [47]. Full genomic and transcriptomic sequences of *Mus musculus* (version GRCm38/mm10) were downloaded from Ensembl [48]. Each sample was aligned against the reference genome using STAR [49] with the additional parameters --outSAMstrandField intronMotif and --quantMode Transcriptome SAM.

### 4.3. Differential Gene Expression Analysis

The alignments obtained with STAR were sorted using samtools software [23]. Once sorted, the matrices with the gene counts were extracted from the aligned reads using HTSeq-count [50] and joined together into a single matrix with custom scripts for downstream expression analyses. Prior to the analysis, we discarded the genes with less than two reads in at least three samples. The subsequent differential gene expression analysis was performed using the DESeq2 v3.20.0 Bioconductor-R package [51], which performs independent filtering of weakly expressed genes, to improve the sensitivity of differential expression tests. Genes with adjusted *p*-values below 0.01 were considered as differentially expressed.

### 4.4. Differential Isoform Expression Analysis

Raw transcript counts were extracted from the transcriptome alignment files using RSEM software v3.3.3 (https://deweylab.github.io/RSEM/) [23]. Then, differentially expressed isoforms were calculated using EBSeq package v3.24.0 [52]. Differential isoform usage between WT mice and *Zrsr1^mu^* mice was considered when their FDR was below 0.01.

### 4.5. Differential Alternative Splicing Analysis and GO Enrichment Analysis

The levels of inclusion of each transcript needed for differential alternative splicing were determined using vast-tools (https://github.com/vastgroup/vast-tools), normalizing the distribution of each AS event by the overall number of that event in the mouse transcriptome (Mm10 annotation). Then, differentially spliced AS events between the two groups were identified by calculating the difference in their average inclusion levels (ΔPSI), filtering those events with low read coverage. The ΔPSI values of each event were extracted from the inclusion table using custom scripts, and those which were higher than 0.1 were considered as differentially spliced. The events were classified as exon skipping (ES), alternative 3′splice site (3′ss), alternative 5′ splice site (5′ss), intron retention (IR), and microexon (MIC). Intron retention events which correspond to U12 events were determined using custom scripts, combining our results with mouse U12 events obtained from U12db (http://genome.crg.es/cgi-bin/u12db/u12db.cgi), to check the U12 enrichment in the *Zrsr1^mu^* mice. Fisher testing was performed to determine the enrichment of each category in relation to the total number of events in the vast-tools database.

Gene Ontology enrichment analysis was performed using the David Gene Functional Classification Tool [53]. Terms with *p*-values below 0.05 were considered as significantly enriched. All the bioinformatics and statistical analyses were performed on the *Finisterrae* server (Supercomputing Center of Galicia).

### 4.6. Western Blotting

Samples from the hypothalami of WT and *Zrsr1^mu^* mice were lysed in 300 μL of RIPA buffer containing 50 mM TrisHCl pH 7.6, 150 mM NaCl, 1% Triton X-100, 0.5% sodium deoxycholate, and 0.1% SDS supplemented with cOmplete^TM^, EDTA-free Protease Inhibitor Cocktail (Roche, Basel, Switzerland) for 1 h at 4 °C. The lysate was centrifuged (12,000*g*; 15 min) and the supernatant collected for protein analysis. Total protein was quantified using the Pierce^TM^ BCA Protein Assay Kit (TermoFisher Scientific, Waltham, MA, USA) following the manufacturer’s instructions. Proteins were resolved by SDS-PAGE (10% of acrylamide loading 50 µg of total protein per well) and transferred onto a nitrocellulose membrane for immunoblotting following standard procedures. Blocking was conducted with 3% BSA in PBS-T, and membranes were incubated overnight at 4 °C with a rabbit polyclonal anti-ZRSR1 antibody, generated against KLH-conjugated synthetic peptide #3: CALEAPPEEDDDVSAN (ChinaPeptides) of the mouse protein (kindly provided by Dr Juan Valcárcel, ICREA and Center for Genomic Regulation (CRG)). Incubation with the secondary antibody goat anti-rabbit IgG-HRP (Santa Cruz Biotechnology, Dallas, TX, USA, sc-2004) was conducted for 2 h at room temperature. The monoclonal anti-β-actin−peroxidase antibody produced in mouse (Merk, Darmstadt, Germany, A3854) was used as the loading control, and blocked membranes were incubated at room temperature for 2 h before development. To develop the plates, we used Amersham ECL Prime Western Blotting Detection Reagent (GE Healthcare Life Sciences, Buckinghamshire, United Kingdom) following the manufacturer’s instructions. The chemiluminescent signal was digitalized using an ImageQuant LAS 500 chemiluminescence CCD camera (GE Healthcare Life Sciences, USA, 29005063). As controls, we employed two HEK293 stable cell lines expressing mouse Zrsr1 (WT) and mouse Zrsr1 truncated protein (ZRSR1mu3) when inducing with tetracycline [1]. Protein extraction and Western blotting in these cell lines were conducted as described for samples from mouse hypothalamus.

### 4.7. Open Field

All animal studies were done with animals of 5–6 months of age. The testing order was the open field first, the elevated plus maze second, and the social interaction test third. Animals were left undisturbed for at least one week between testing procedures. The open field test [54] was performed in a black wooden box (40 × 40 × 45 cm high), under constant illumination, and the motor activity was recorded and analyzed using SMART video tracking software (Panlab, Llobregat, Barcelona, Spain). Animals were placed in the center of the arena and their exploratory activity was monitored for 5 min. Then, the animal was picked up and the area was cleaned with a 5% ethanol solution.

### 4.8. Elevated Plus Maze

The elevated plus maze [55] consisted of two opposing open arms, 50 × 10 cm, and two enclosed arms, 50 × 10 × 40 cm, made of wood, elevated 50 cm above the floor and with the central square formed by the arms left open. The experiment took place in a sound-isolated room illuminated by a dim light. The mouse was positioned in the central square, and the event was video recorded and analyzed using SMART video tracking software (Panlab); after 5 min, the animal was returned to its home cage and the apparatus was cleaned. The time spent in the open arms of the maze was considered an index of the animal’s anxiety.

### 4.9. Social Behavior

The general design of the social interaction test was adapted from [56]. A pair of mice confronted each other in a familiar area, weakly lit by a red 40 W bulb. The social encounters were videotaped and posteriorly quantified using JWatcher 1.0 by experienced researchers blind to the experimental conditions [57]. The following behavioral categories were considered: body care, digging, nonsocial exploration, exploration from a distance, social investigation, threat, attack, avoidance/flee, defense/submission, and immobility.

### 4.10. Proliferation and Survival Analysis in the Hypothalamus and Subventricular Zone

5′-bromo-2′-deoxyuridine (BrdU, cat. no. B5002, Sigma, St. Louis, MO, USA) was dissolved at 15 mg/mL in sterile 0.9% NaCl solution. BrdU was intraperitoneally (i.p.) administered at a dose of 50 mg/kg body weight twice per day for three consecutive days (Days 1–3, 6–8 h between injections). 5′-iodo-2′-deoxyuridine (IdU, cat. no. I7125, Sigma) was also dissolved at 15 mg/mL in sterile 0.9% NaCl solution. IdU was i.p. administered at a dose of 57.65 mg/kg body weight twice per day for three consecutive days (Days 17–19, 6–8 h between injections). The animals were killed 12 h after the last injection of IdU was administered.

#### 4.10.1. Brain Collection

All animals were i.p. anesthetized (sodium pentobarbital, 50 mg/kg body weight) and transcardially perfused with 4% formaldehyde in 0.1 M phosphate buffer (PB). The brains were dissected out and kept in the same fixative solution overnight at 4 °C. The brains were then cryoprotected and cut into 30 μm thick coronal sections by using a sliding microtome (Leica VT1000S). Sections were divided into five parallel series until use for immunohistochemistry.

#### 4.10.2. Immunohistochemistry

Free-floating coronal sections from −1.58 to −2.46 mm Bregma levels (hippocampus and hypothalamus) and 1.42 to −0.10 mm Bregma levels (striatum) from one of the five parallel series obtained from each mouse brain were selected for each immunohistochemistry analysis (Mouse Atlas, [58]). Sections were first washed several times with PBS to remove sodium azide. Then, sections were incubated in a solution of 3% hydrogen peroxide and 10% methanol in PB 0.1 M for 45 min at room temperature in darkness to inactivate endogenous peroxidase. After three washes in PBS for 10 min, DNA was denatured by exposing sections to HCl 2 N for 15 min at 37 °C, followed by two washes in borate buffer 0.15 M to neutralize pH. After three additional washes in PBS for 10 min, sections were incubated in a blocking solution containing 10% donkey serum, 0.3% Triton X-100, and 0.05% sodium azide in PBS for 45 min. Sections were incubated overnight in the primary antibody rat anti-BrdU (1:500; Accurate Chemical & Scientific, Westbury, NY, USA, cat. no. OBT0030G) at 4 °C. For IdU immunohistochemistry, brain sections were firstly incubated in a solution of 3% hydrogen peroxide and 10% methanol in PB 0.1 M for 10 min; secondly incubated in a blocking solution containing 5% goat serum, 0.3% Triton X-100, and 0.05% sodium azide in PBS for 1 h; and finally incubated overnight in the primary antibody mouse anti-IdU (1:500; Rockland, Gilbertville, PA, USA, cat. no. 200-301-H51) at room temperature. The following day the sections were incubated in the biotinylated donkey anti-rat IgG (H+L) antibody (1:500, Novex; cat. no. A18743) or the biotinylated goat anti-mouse IgG antibody (1:1000, Sigma; cat. no. B7254) for 90 min. The sections were then incubated in ExtrAvidin peroxidase (Sigma, St. Louis, MO, USA) diluted 1:2000 in darkness at room temperature for 1 h. Finally, immunolabeling was revealed with 0.05% diaminobenzidine (DAB; Sigma) and 0.03% H_2_O_2_ in PBS.

#### 4.10.3. Quantification of BrdU- and IdU-Immunoreactive Cells

The average density of positive cells per animal was quantified. Thus, the estimation of the number of cells per section (30 μm deep) in both hemispheres was calculated. Each structure analyzed consisted of ~6 coronal sections, which resulted in one of every five equidistant sections (one representative section for each 180 μm) according to the rostro-caudal extent. To outline the area of study, the region of interest was drawn in each structure, the identification of which was performed at Bregma −1.58 to −2.46 mm in hippocampal and hypothalamic levels and at Bregma 1.42 to −0.10 mm in striatal levels according to a mouse brain atlas and cytoarchitectonic criteria [58]. Thus, the BrdU-immunoreactive (+) nuclei and IdU-immunoreactive (+) nuclei that came into focus were manually counted in the hypothalamus and the subventricular zone (SVZ) of the lateral ventricles. Regarding the hypothalamus, counting was performed in the ventromedial (VMH) and arcuate (ARC) nuclei of the hypothalamus and median eminence. Quantification was performed using a standard optical microscope with 40× objective (Nikon Instruments Europe B.V., Amstelveen, The Netherlands) coupled to NIS-Elements Imaging Software 3.00 (Nikon). The data were expressed as the number of positive cells per section.

## Figures and Tables

**Table 1:** Differentially spliced events detected in the *Zrsr1^mu^* mice.

Event Type	*Zrsr1^mu^* Up	*Zrsr1^mu^* Down	Total Events (Vast-Tools)	Enrichment (*p*-Value)
3SS	165	186	7048	0.0906
5SS	182	181	7101	0.517
ES	444	346	20,935	2.20 × 10^−16^
MIC	8	5	643	3.82 × 10^−4^
IR (U12)	665 (98)	276 (17)	13,903	2.20 × 10^−16^
Total	1464	994	49,630	

## Supplementary Materials

Supplementary materials can be found at https://www.mdpi.com/1422-0067/20/14/3543/s1. Supplementary Table S1. Summary of RNA-seq alignment; Supplementary Table S2. Differentially expressed genes in Zrsr1mu and WT mouse hypothalami; Supplementary Table S3. Differentially expressed isoforms in Zrsr1mu and WT mouse hypothalami; Supplementary Table S4. List of common up-regulated and down-regulated genes and isoforms; Supplementary Table S5. Gene Ontology analysis of up- and down-regulated genes in Zrsr1mu hypothalamus; Supplementary Table S6. List of alternative splicing events in Zrsr1mu and WT mouse hypothalami; Supplementary Table S7. Gene Ontology analysis of preferentially retained U12 introns in Zrsr1mu vs. WT mouse hypothalami.
